# Allogeneic Hematopoietic Stem Cell Transplantation for Infant Leukemia: A Single-Center Case Series and Narrative Review

**DOI:** 10.3390/children12101418

**Published:** 2025-10-20

**Authors:** Irina Kostareva, Kirill Kirgizov, Irina Shubina, Nara Stepanyan, Nataliya Batmanova, Timur Valiev, Mihail Kiselevsky, Svetlana Varfolomeeva

**Affiliations:** 1 Research Institute of Pediatric Oncology and Hematology, FSBI “N.N. Blokhin National Medical Research Center of Oncology” of the Ministry of Health of Russia, Kashirskoye sh. 24, Moscow 115522, Russia; k.kirgizov@ronc.ru (K.K.); nara19922@yandex.ru (N.S.); batmanova_nataly@mail.ru (N.B.); timurvaliev@mail.ru (T.V.);; 2 Research Institute of Experimental Diagnostics and Therapy of Tumors, FSBI “N.N. Blokhin National Medical Research Center of Oncology” of the Ministry of Health of Russia, Kashirskoye sh. 24, Moscow 115522, Russia; irinashubina@mail.ru (I.S.); kisele@inbox.ru (M.K.)

**Keywords:** hematopoietic stem cell transplantation, acute leukemias, infant leukemia, treatment, diagnostic

## Abstract

Background/Objectives: Acute leukemias (AL) in children under 1-year-old are combined under the term “infant leukemia” and are a very rare malignancies, accounting for up to 5% of all childhood AL cases. The predominance of unfavorable clinical and laboratory characteristics leads to unsatisfactory treatment results, even with the use of modern treatment protocols. Patients/Methods: A comprehensive search through MEDLINE, PubMed, Scopus, and ScienceDirect using infant leukemia-related keywords was performed and included a final set of 52 academic articles. Our own experience included 11 patients with infant leukemia underwent allo-HSCT (allogeneic hematopoietic stem cell transplantation) at the NN Blokhin National Medical Research Center of Oncology in 2021–2023. Types of leukemia included acute myeloid leukemia, lymphoblastic leukemia, and mixed-phenotype acute leukemia. The most frequent cytogenetic aberration was *KMT2A*. All patients were in clinical and hematological remission, but four had positive MRD status (minimal residual disease). Donors: haploidentical—5 (45.4%), matched unrelated donor—5 (45.4%), and matched related donor—1 (9.2%). Graft manipulations: post-transplant cyclophosphamide was given to three patients with haplo-HSCT, and TCRαβ/CD19 depletion was performed in two patients. The type of immunosuppressive therapy (IST) varied based on the donor. Conditioning regimens were myeloablative. Results: Median follow-up was 23.5 months. Acute GVHD grade I–II developed in two patients (18%) and grade III–IV in three patients (27%). The overall survival rate was 54.5% (n = 6). The relapse rate after allo-HSCT was 18% (n = 2). The most common cause of treatment failure was infectious complications in the early post-transplant period (70%). Conclusions: Our center’s experience demonstrated acceptable transplant-related mortality and satisfactory relapse rates after allo-HSCT in patients with infant leukemia. The treatment of acute leukemia in infants is challenging, and optimal protocols are being developed around the world specifically for these patients. Taking into account the characteristics of this age group, the choice of chemotherapy drug doses should be carefully considered, and the indications for allo-HSCT should be balanced.

## 1. Introduction

Infant acute leukemia (IL) is a subset of malignancies affecting children under one year of age at diagnosis. The average incidence of IL in the United States is 41 cases per 1 million newborns per year [1]. Global statistics show a significant increase in the incidence of acute lymphoblastic leukemia (ALL) and acute myeloid leukemia (AML) in infants between 1975 and 1989, approximately 2.5% per year [2]. The incidence of ALL in infants is much lower than in children aged 1 to 14 years and is about the same as in adolescents. In contrast, the incidence of AML in infants is about twice as high as in older children and adolescents. The ratio of ALL/AML is about 60% and 40%, respectively [1,2,3]. This group of patients is considered to have the worst survival rates and prognostic factors. It accounts for 1% of all pediatric ALL cases. Most cases are B-lineage ALL, while T-lineage and mixed-phenotype (MPAL) represent a small percentage. The age of the patient is important for the prognosis of survival in pediatric leukemia. In contrast to AML, ALL is characterized by poorer outcomes in infants compared to older children with similar cytogenetic characteristics. In addition, pediatric leukemias are characterized by aggressive symptoms, high-risk cytogenetic abnormalities associated with resistance to chemotherapy, high relapse rates, and increased toxicity and long-term effects of therapy [2,3]. In particular, *KMT2A*-rearranged ALL types are characterized by hyperleukocytosis, a relatively high incidence of central nervous system (CNS) involvement, an aggressive course, and early relapses leading to a poor prognosis. Researchers have observed that different types of rearrangements in the *KMT2A* gene, very high leukocyte count, age less than six months, and poor response to prednisone prophase are independent poor prognostic factors [4,5]. Cytogenetic analysis plays a key role in diagnosing and assessing risk in infant leukemia. *KMT2A* rearrangements in children with AML— including monocytic (FAB M5), myelomonocytic (FAB M4), and megakaryoblastic (FAB M7) subtypes—can affect the prognosis in different ways, depending on the specific partner gene. Testing for megakaryocytic markers in all cases of infant leukemia is important because diagnosing acute megakaryoblastic leukemia can be difficult. Additionally, transient abnormal myelopoiesis, which resembles acute megakaryoblastic leukemia in both morphology and immunophenotype, frequently affects infants with Down syndrome and is characterized by a unique somatic mutation in *GATA1* [6]. Although the pathogenesis of IL in children younger than one year of age is unclear, there is no doubt that the onset usually occurs in utero and is strongly associated with the presence of predisposing genes as well as leukemogenic exogenous exposure (chemicals, ionizing rays) [1,4,7]. During the early stages of pregnancy, the growing fetus is more sensitive to the effects of potential DNA damage. The etiology of acute leukemia in infants with *KTM2A-r* may be related to transplacental exposure to DNA topoisomerase inhibitors. Topoisomerase inhibitors include chemotherapeutics and benzene metabolites such as benzoquinones, isoflavones, flavonoids, lignans, podophyllin resins, quinolone antibiotics, and some pesticides [1,7].

Recent international studies (e.g., Interfant Study Group, Children’s Oncology Group protocols) have shown that the three-year overall survival rate for infants with ALL following allogeneic HSCT is approximately 40–60%. However, transplantation is discussed more frequently in patients with adverse cytogenetic variants, particularly those with *KMT2A/AFF1* genetic alterations and a poor response to initial treatment.

In some cases, current chemotherapeutic protocols show similar results to those of transplantation, even in patients with an aggressive disease course, especially in infants without extremely high-risk factors. International practices show that the decision for allo-HSCT is made on a case-by-case basis in a multidisciplinary consultation involving transplant specialists, oncologists, and pediatricians, taking into account the response to treatment by MRD and the cytogenetic characteristics of IL.

## 2. Materials and Methods

**Figure 1:**
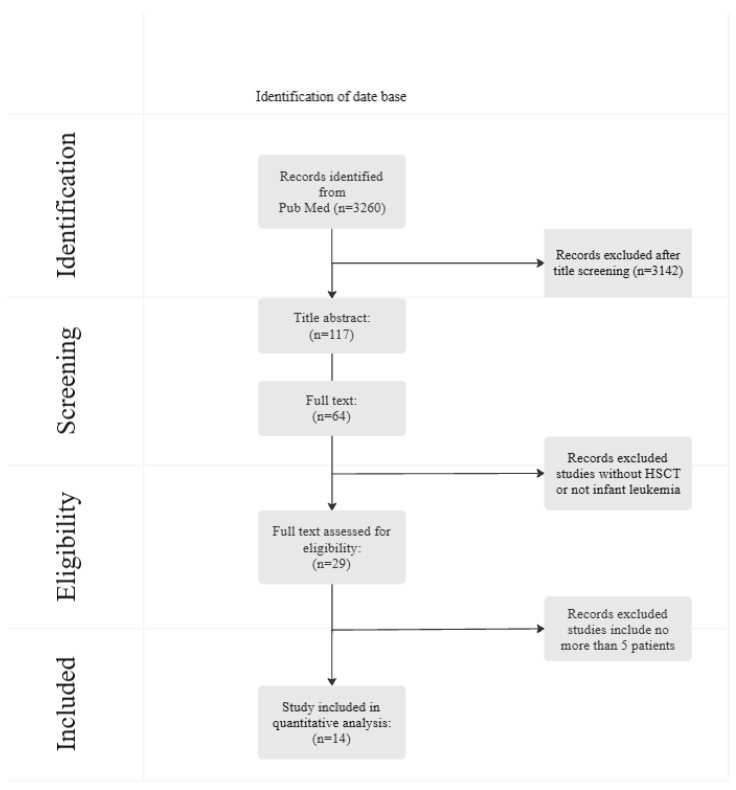
Flow chart for selection of studies.

A comprehensive search was performed through MEDLINE, PubMed, Scopus, and ScienceDirect using the following keywords: “hematopoietic stem cell transplantation”, “infant leukemia”, “treatment”, “diagnostic”, and “clinical trial” in the title and abstract, and in the sections of review, systematic review, meta-analysis, clinical trial, and randomized clinical trials. We opted for articles published within the last 10 years in the English language. Of the articles that emerged, the most recent ones were selected, especially those that focused on diagnostic approaches in patients with IL published over the last decade. The final set included 64 academic articles related to the topics of interest. Fourteen articles devoted to clinical studies of allo-HSC in the treatment of IL were selected, (Figure 1).

At the NN Blokhin National Medical Research Center of Oncology (Moscow), in 2021–2023, eleven patients with infant leukemia underwent allo-HSCT (Table 1). In the study, the median age at diagnosis was 5.7 months (0 to 11 months). There were six (54.5%) patients with AML, three (27.2%) patients with ALL, and two (18.3%) infants with mixed-phenotype acute leukemia. *KMT2A* gene rearrangements were observed in six (54.5%) patients. The median time from diagnosis to allo-HSCT was 6 months (from 3 to 11 months). Patients were primary treated according to the protocols: AML-BFM 2004, AML-MRD 2018, and ALL-IC BFM 2009. Gender distribution was as follows: five girls and six boys. Donors were equally distributed: haploidentical (MMRD)—5 (45.4%), matched unrelated donor (MUD)—5 (45.4%), and one patient had matched related donor (MaRD)—1 (9.2%). The transplantation sources used were bone marrow—2 pts and peripheral blood stem cells—9 pts. The median cell dose infused was 3.0 × 10^8^ total nucleated cells per kilogram. Post-transplant cyclophosphamide was administered to three patients with haplo-HSCT, and TCRαβ/CD19 depletion was performed in two patients. Conditioning regimens were myeloablative with reduced toxicity and included the cumulative doses: fludarabine 120–150 mg/m^2^, treosulfan 36 g/m^2^ or busulfan 12 mg/kg, thiotepa 5–10 mg/kg or melfalan 100–120 mg/m^2^. One patient with refractory T-ALL received busulfan 12 mg/kg, cytarabine 4000 mg/m^2^, and cyclophosphamide 100 mg/kg as part of the conditioning regimen. Drug doses were calculated per square meter regardless of the child’s weight. Patients with TCRαβ/CD19 did not receive standard immunosuppressive therapy (IST) for prevention of graft-versus-host reactions (they received rituximab/tocilizumab/abatacept alone on day −1). In GVHD prophylaxis with post-transplant cyclophosphamide or in MaRD transplants, the basic IST includes cyclosporine A or ruxolitinib, starting from day −1 of conditioning (approximately up to 100 days). In MUD, GVHD prophylaxis included antithymocyte immunoglobulin (ATG), and tacrolimus or ruxolitinib is prescribed as the basic IST. Regardless of donor type, all patients receive rituximab and abatacept on day −1 of conditioning.

## 3. Diagnosis

### 3.1. Genetics

The most common form of IL is characterized by cytogenetically balanced chromosomal translocations that include the mixed-lineage leukemia gene (*KMT2A* gene previously named as *MLL*) on chromosome 11q23, first described in 1991–1992 [5,7]. Fetal and neonatal hematopoietic progenitor cells may be particularly susceptible to the effects of *KMT2A* fusion proteins. Women and men have similar frequencies of *KMT2A* rearrangements (*KMT2A-r*). *KMT2A-r* occur in up to 5% of ALL cases in children of all ages with a predominance of 70 to 80% in infant ALL. *KMT2A-r* in AML is most common in the group of IL—50%. *KMT2A* rearrangements result in fusion of *KMT2A* (11q23.3) to partner genes [8]. To date, more than 80 different *KMT2A* partner genes have been identified. In infant ALL, four partner genes account for 93% of cases: *AFF1* (49%), *ENL* (22%), *AF9* (17%), and *AF10* (5%) [8]. *KMT2A-r* in infants with ALL has a well-defined gene expression profile. One of the features is the overexpression of *FLT3*. *FLT3* signaling in these cases either by activating mutations or, more commonly, by autocrine activation through co-expression of the ligand *FLT3* [9]. *FLT3* overexpression has been shown to have an unfavorable prognostic, especially in children under one year of age with *KMT2A-r* ALL [9,10]. The specific expression of chondroitin sulfate proteoglycan-4, also known as neutron glial antigen-2 (NG2), is also characteristic of *KMT2A-r* ALL [9,10,11]. It is a transmembrane proteoglycan that has very low expression in normal hematopoietic cells. *NG2* expression is common in *KMT2A-r* ALL (about 90% of cases) [12]. *NG2* has been the subject of much recent investigation and has become a novel therapeutic target for *KMT2A-r* ALL due to its predictive value, because it contributes to leukemia invasiveness and CNS infiltration, and more frequent CNS relapse [13,14,15]. *KMT2A-r* are acquired in hematopoietic progenitor cells in the fetus, leading to rapid progression and clinical manifestations of IL [12,13,14,15]. *KMT2A-r* in AML is associated with the monocytic differentiation of AML. Dysregulation of *HOX* genes is a common feature of AML [14]. *HOX* genes play a key role in the regulation of hematopoietic development. Dysregulated *HOX* gene expression can result from chromosomal translocations involving upstream regulators such as *KMT2A*. Specific clinical trials for infant ALL are conducted by three major cooperative groups: Interfant (based in Europe), COG (based in North America), and the Japanese Pediatric Leukemia Study Group. All recently completed trials have used a prospective, risk-stratified approach including *KMT2A-r* status and age. In contrast to infants, patients older than one year have more favorable genetic features, in the form of high hyperdiploidy and *ETV6::RUNX1* fusion. Studies have shown that older children share cytogenetic abnormalities with *KMT2A-r*, albeit with a different distribution; the proportion of patients with favorable genetic risk (hyperdiploidy, *ETV6::RUNX1*) is higher (60% vs. 12% *KMT2A-r*). Next-generation sequencing (NGS), involving DNA, RNA, or miRNA sequencing, provides a tool for identifying the most important alterations in ALL that may help determine the prognosis and pathogenesis [14].

### 3.2. Clinical Features

**Figure 2:**
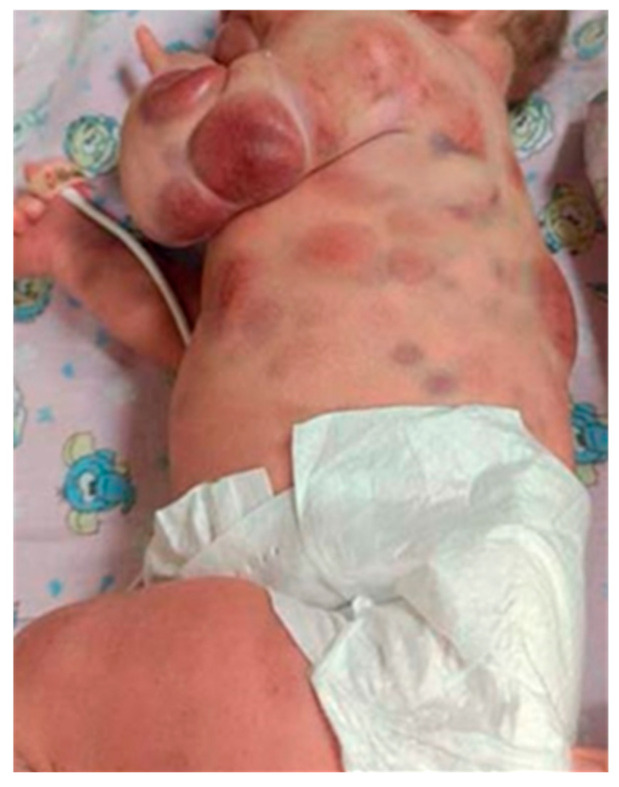
Leukemoid skin infiltration (own clinical case).

Acute childhood leukemias often presents with typical leukemia symptoms such as bruising, bleeding, fever, asthenia, and hepatosplenomegaly. As for patients with infant leukemia, they are characterized by more aggressive manifestations and early central nervous system involvement [15]. Thus, children under one year of age typically show higher initial hyperleukocytosis and a greater risk of tumor lysis syndrome (TLS). In addition, IL often presents with skin infiltration (Blueberry Muffin Syndrome) and other extramedullary lesions (Figure 2). Leukemoid skin infiltration is more characteristic of myeloid IL and occurs in about two-thirds of patients (common in FAB-M4 and FAB-M5 AML), whereas it is rare in lymphoblastic IL [10,15]. They are unique in that they can occur without peripheral blood or bone marrow involvement. Leukemoid skin infiltration is described as multiple papules and nodules of blue, red, or brown color. This type of leukemia has a very poor prognosis. It is possible that chemotherapy may not be able to penetrate the skin sufficiently, resulting in a higher relapse rate in these patients.

These different clinical features of IL may be caused by malignant transformation in different stages of fetal hematopoiesis. Also, the peculiarities of IL can be attributed to the transformation of one leukemia type into another, heavier tolerance of chemotherapy, and more severe late complications of therapy (endocrine, cardiovascular, neurologic).

### 3.3. Instrumental and Laboratory Diagnostics

As mentioned above, IL is characterized by initially higher leukocytosis, reaching values with a WBC count > 400,000 cells/µL. Acute leukemia is diagnosed when more than 20% of blast cells are present in a bone marrow puncture. There are specific morphological and immunophenotypic features in IL. FAB (French–American–British classification) types more characteristic of IL include M4, M7 (megakaryoblastic), and M5 variant (monoblastic) [16]. Lymphoblastic IL is more likely to have the L1 subtype than the L2 subtype and less likely to have the L3 subtype [10,16]. Immunophenotypic characterization of infant ALL and AML are distinct: *KMT2A-r* ALL is CD19- positive/CD10 negative and often co-express one or more myeloid antigens, suggesting that they are of the nature of immature lymphoid forerunners [10]. Infant leukemia patients may have an undefined mixed phenotype (mixed-phenotype acute leukemia) or lack differentiation markers (acute undifferentiated leukemia). In the cytological examination of biopsy specimens from skin infiltrates, the presence of blast cells confirms the diagnosis of leukemia, even in the absence of blast cells in the peripheral blood [16,17]. Abdominal ultrasound reveals marked hepatosplenomegaly; the lower edge of the liver may reach the pelvic cavity.

## 4. Treatment

The use of ALL and AML chemotherapy regimens or specific protocols for IL (Interfant-99/06), with drug doses calculated per kilogram of body weight, is unique in the treatment of IL. The use of intensive chemotherapy as well as allo-HSCT is not excluded by functional features of organs in children under one year of age. One of the challenging aspects of treating IL is the increased incidence of toxic and infectious complications during chemotherapy, which can be explained by the complexity of physiological processes during the first year of life [16,17,18].

There are currently three major groups focused on conducting clinical trials specific to IL: the Children’s Oncology Group (COG), the Japanese Pediatric Leukemia/Lymphoma Study Group (JPLSG), and the Interfant Study Group [19,20].

The Interfant Study Group is the largest international organization that conducts research activities in the field of IL, involving various international research groups, including the BFM group. Their study included 482 infants divided into standard and high-risk groups (based on response to one week of systemic prednisolone) between 1999 and 2005. Factors associated with an adverse outcome included: age less than six months, *KMT2A-r*, poor response to weekly prednisolone prophase, CD10 negativity, and initial hyperleukocytosis [21]. The two-year treatment protocol was based on a hybrid regimen that included elements used to treat both ALL and AML, while minimizing the use of anthracyclines and alkylating agents. For high-risk patients, allo-HSCT was only considered if a compatible donor was available. Higher MRD (minimal residual disease) levels at the end of induction and consolidation were significantly associated with poorer disease-free survival. These studies demonstrated that MRD is an important prognostic factor and that its diagnosis adds value in identifying risk groups in children with ALL. MRD analysis may also be useful in the decision-making process regarding allo-HSCT. Subsequently, another study was conducted in the group named Interfant-06, which differed from its predecessor by removing dexamethasone and vincristine during maintenance chemotherapy. However, the new treatment approach did not significantly improve outcomes for children with ALL—there was no significant difference in 6-year EFS when compared (46.1% in both protocols).

The COG trial, known as P9407, was designed to provide shortened but intensified therapy by eliminating age- and weight-based dose reductions for most chemotherapy drugs to improve EFS. In the P9407 results, toxicity mortality (mainly infectious) in the first 90 days of treatment was reported in 25% of 68 infants. After the study was modified—prednisolone was changed to dexamethasone, and the dose of daunorubicin was reduced—the early mortality rate dropped to 6% [22].

The Japanese Pediatric Leukemia/Lymphoma Study Group combined two protocols for the treatment of infant leukemia, named MLL96 and MLL98. Between 1995 and 2001, 102 patients with IL, with and without *KMT2A-r*, were included [23]. Patients with *KMT2A-r* in first remission after intensive chemotherapy were to receive allo-HCT from any available donor, but this approach revealed a high incidence of early relapses. These studies demonstrated the benefits of risk-adapted therapy according to *KMT2A* status, with outcomes significantly better in patients without *KMT2A* gene involvement, 5-year EFS and OS of 95.5% [23,24]. The 5-year EFS and OS in patients with *KMT2A* were 38.6% and 50.8%, respectively.

In the analysis of the BFM treatment protocols BFM-98 and BFM-2004 for infant AML, the EFS was 43% in the *KMT2A-r* group and 52% in the group without *KMT2A-r* [24,25]. The Japan Children’s Cancer Group in 2003 showed the results of treating infant AML with intensive chemotherapy alone and analyzed prognostic factors. Thirty-five patients with infant AML treated with intensive chemotherapy alone between 1995 and 1998 were included in this study. Induction therapy included etoposide, cytarabine, and mitoxantrone. Four different courses of intensification therapy were then used, including etoposide, cytarabine, and anthracyclines or vincristine. According to the results, 3-year OS and EFS were 76% and 72%, respectively. The study also showed that MRD level by flow cytometry after consolidation therapy can be a predictor of AML outcomes [26].

Also, individual case studies on treatment outcomes in acute myeloid IL have been reported in the literature. For example, in 2009, a group of European researchers reported their experience in treating a patient with the M6 variant of infant acute myeloid leukemia. The treatment was carried out according to the MRC-12 protocol with age adjustment. Complete remission was achieved after one course of ADE (cytarabine, daunorubicin, etoposide). Treatment was complicated by several episodes of febrile neutropenia and infections. The patient has since been in durable complete remission for four years [10,27].

Modern genetic studies are opening new avenues in the treatment of *KMT2A-r* IL through the introduction of new targeted drugs. These are the most commonly used drugs: proteasome inhibitors, hypomethylating agents (such as cytosine analogs azacytidine or decitabine), menin-*KMT2A* inhibitor, *FLT3* inhibitors, curaxin CBL0137, histone deacetylase inhibitors, and CAR- T-cell [16,17]. The use of monoclonal antibodies such as blinatumomab and inotuzomab ozogamicin are also actively used in the treatment of patients with ALL across different age groups, including infants.

### Role and Place of Allo-HSCT

The need for allo-HSCT in IL (particularly AML) remains controversial. The use of allo-HSCT in IL is always a subject of discussion due to contradictory data and peculiarities of this age category. On the one hand, a number of studies and guidelines point to the high aggressiveness and unfavorable prognosis of leukemia in infants, especially in the presence of *KMT2A-r*, which justifies the early use of allo-HSCT even in the first remission. Arguments in favor of early allo-HSCT include the potential to achieve long-term remission, reduced risk of relapse, and data from individual protocols where survival was higher in transplanted patients.

On the other hand, critics draw attention to the extremely high risks of the procedure itself in children in the first year of life: unfavorable tolerability, high incidence of complications (infections, GVHD, organ failure), and transplant-related mortality risks. In addition, modern intensive chemotherapeutic protocols with optimal supportive care can already achieve remission in some patients, and the potential benefits of HSCT do not always outweigh the risks, especially in the absence of unfavorable genetic markers.

In 2015, JPLSG published the results of treating 62 children with *KMT2A-r* ALL by a short course of intensive chemotherapy followed by early allo-HSCT for four months. The EFS and OS were 43.2% and 67.2%, respectively, which was significantly higher than in the group of patients who received chemotherapy alone [28].

The results of the Interfant-99 study, which included 483 children with previously untreated ALL between 1999 and 2006, showed that allo-HSCT in first remission is a possible treatment strategy for intermediate-risk infant *KMT2A-r* ALL with high MRD level [13]. All patients were stratified into two arms based on response to a 1-week prednisolone phase. All patients received a 5-week induction regimen (prednisolone, dexamethasone, vincristine, daunorubicin, L-asparaginase, low-dose cytarabine, and CNS prophylaxis) followed by a 4-week consolidation regimen (high-dose methotrexate, 6-mercaptopurine, high-dose cytarabine, L-asparaginase, and CNS prophylaxis) and seven weeks of reinduction therapy (dexamethasone, 6-thioguanine, vincristine, daunorubicin, low-dose cytarabine, cyclophosphamide, and CNS prophylaxis) [29]. Patients with a poor response to the 7-day prednisolone group were indicated for allo-HSCT. A study of 297 patients with *KMT2A-r* in first remission found a statistically significant difference in DFS. After adjusting the waiting time for transplant, of the 277 patients (93%), 37 (13%) underwent allo-HSCT, while 240 patients (87%) received chemotherapy alone. The 5-year OS rates were 65.6% in the allo-HSCT group and 48.6% in the chemotherapy group. The 5-year DFS rates were 60.1% in the allo-HSCT group and 46.8% in the chemotherapy group.

The BFM group recommended etoposide, busulfan, and cyclophosphamide as the conditioning regimen. Meanwhile, total body irradiation (TBI) was strongly discouraged due to concerns about long-term side effects. Cyclosporine A was used for GVHD prophylaxis [29]. At a median follow-up of 5 years, the EFS rate for *KMT2A-r* ALL was only 38.6%, but this was an improvement compared with previous studies. Furthermore, the results of a study using TBI during the conditioning regimen showed that, of 14 infants with *KMT2A-r* ALL transplanted in first remission, 78.5% survived, and only moderate delayed endocrine and nervous system effects were observed [29,30].

More recently, the results of the Children’s Cancer Group (CCG 1953) and Pediatric Oncology Group (POG 9407) studies on the treatment of infant ALL were presented, using similar chemotherapy regimens and recommending allo-HSCT in *KMT2A-r* cases [31].The 5-year EFS rates were 50.9% in the transplant group and 48.7% in the non-transplant group, suggesting no clinical benefit from the first remission of allo-HSCT in infants with *KMT2A-r* ALL [32,33].

The effect of allo-HSCT in infant AML is also still unclear, due to the lack of randomized trials. In early studies, allo-HSCT provided long-term survival in some cases of AML among infants. The Japan Children’s Cancer Group analysis included all cases of AML in infants less than two years of age treated between 1974 and 1995 [34]. The conditioning regimen and GVHD prophylaxis differed among patients. It has been shown that infants with AML receiving allo-HSCT had a 5-year disease-free survival rate of 73% [34,35]. However, allo-HSCT was used in only four patients, while the others received autologous HSCT.

The Pediatric Oncology Group also compared the effect of intensive induction chemotherapy followed by allogeneic or autologous HSCT for infants with AML—survival rates in the allo-HSCT and chemotherapy groups were better than in the autologous HSCT group (71% vs. 40%) [36]. It was demonstrated that a conditioning regimen containing busulfan, melphalan, and cyclophosphamide was most commonly used for infants with AML and was well tolerated, avoiding serious long-term effects [35,36]. The largest and longest clinical study on the efficacy of allo-HSCT included 2498 infants with malignant or non-malignant diseases. A 15-year follow-up allowed the authors to conclude that during this observation period, there was no increase in survival rates among infants with malignant diseases. A high relapse rate in this group and toxicity remain serious challenges in the application of allo-HSCT [37].

A recent Chinese study involved 27 infants with AML who underwent allo-HSCT. Peripheral blood and bone marrow, as well as umbilical cord blood, were used as sources of stem cells. No significant differences between the groups were noted in terms of efficacy and the development of GVHD; however, thrombopoiesis recovery was slower in the umbilical cord blood allo-HSCT group. The authors emphasized the importance of MRD status. Specifically, among patients with negative MRD, only one of these patients experienced a relapse, compared to the group with MRD-positive results [38].

Table 2 shows some more studies in the field of treatment of infant leukemia using allo-HSCT.

Differences in the results of research studies have led to discrepancies between research groups regarding the role of allo-HSCT in current infant protocols.

## 5. Results

Median follow-up was 23.5 months (IQR 11–47.2). Table 3 shows our outcomes of allo-HSCT patients with IL.

Engraftment. Recovery of the granulocytes was recorded when neutrophil counts exceeding 500 cells/µL, with or without G-CSF stimulation. Recovery of the megakaryocytic lineage was considered to have occurred when platelet counts exceeded 20,000/µL for three consecutive days without the need for platelet concentrate replacement [47]. Primary graft failure was recorded in two patients; one of them required a second allo-HSCT and the other died from infection. Nine patients have hematopoietic reconstitution, median time to neutrophil engraftment in group of MUD HSCT was 13 days (range: 12–14) and platelet engraftment was 12 days (range: 11–12). In the haplo-HSCT group, the median time of neutrophil engraftment was 16 days (range: 14–18) and platelet engraftment was 20 days (range: 14–25). In one patient who underwent transplantation from MaRD, neutrophil engraftment was recorded on day 16, and platelet engraftment was recorded on day 12. All patients after allo-HSCT underwent a study of donor chimerism by qPCR in bone marrow on days 30, 100, 180, and 360 after HSCT. In five patients who were alive at the time of the study, complete donor chimerism was determined (total and by CD3+ and CD34+ lines). In one patient with primary graft failure on day +30, total chimerism consisted of 96.5% of their own cells. In one patient with a very early leukemia relapse, chimerism was not determined. A patient with relapse four months after HSCT showed mixed chimerism (donor lymphocyte infusion was performed). Three patients who died due to infectious-immune complications on day 30 showed satisfactory donor chimerism.

GVHD. During our study, the MAGIC 2018 criteria were used to establish the diagnosis of acute GVHD. These criteria were validated by multicenter study data in both children and adults. Staging of chronic GVHD was performed according to the NIH 2017 criteria. Grade I-II acute GVHD developed in two patients (18.1%) and grade III-IV in three patients (27.2%). At the same time, a more severe degree of aGVHD developed in patients who underwent HSCT from MMRD with post-transplant cyclophosphamide administration (n = 2) and in one patient with MUD HSCT; all patients had involvement of the skin and gastrointestinal tract. Despite the administration of multiagent immunosuppressive therapy, these patients died in the early post-transplantation period due to the development of severe infection. Two patients developed severe cGVHD: one patient after MUD HSCT and one after haplo-HSCT with TCRαβ/CD19 depletion. Both patients had a history of GVHD grade I–II. In one patient, cGVHD involved the skin, mucous membranes, joints, and lungs; the second patient had cGVHD affecting the skin and its appendages. Currently, these patients are continuing immunosuppressive treatment and follow-up while being in remission for leukemia. With the introduction of modern methods for treating chronic GVHD, such as extracorporeal photopheresis, mesenchymal stem cells, and new pharmacological agents (ruxolitinib, ibrutinib, axatilimab, belumosudil), post-transplant mortality rates have been successfully reduced. The patients are monitored on an outpatient basis and do not require prolonged hospitalization. However, long-term immunosuppression, including the use of steroids, undoubtedly affects the patient’s development and quality of life.

Survival and relapse outcomes. Following allo-HSCT, remission status was systematically evaluated at four key timepoints (30/100/180/360 days) using a multi-method approach that included bone marrow aspiration, myelogram examination, MRD immunophenotyping analysis, and cytogenetic assessment. In our study, relapse of leukemia occurred in two patients, both of whom died (on days 20 and 120 after allo-HSCT). The relapse rate after allo-HSCT was 18%. The overall survival rate was 54.5% (n = 6), in the group of patients with *KMT2A-r*–60% (n = 3).

The most common cause of treatment failure was aGVHD and infectious complications in the early post-transplant period. There are no serious long-term effects of allo-HSCT; surviving children developed comparably to their peers (with the exception of two patients with cGVHD).

## 6. Discussion

The problems of treating IL are multifaceted. Despite the development of genetic engineering and cellular technologies, the mortality rate of patients with IL remains at a high level. Effective chemotherapy requires dose modification due to factors affecting the pharmacokinetics of drugs differently than in other age groups, which is due to the peculiarity of infants’ hematopoiesis [41]. The toxic effects associated with treatment in both the early and late periods are problems that necessitate reducing chemotherapy doses. Long-term complications that can occur after allo-HSCT in infants are the result of both pre-transplant chemotherapy and immunosuppressive therapy. Many long-term complications in children have been associated with total body irradiation, and despite the fact that this treatment method shows great effectiveness in terms of myeloablation, physicians refrain from using it in children under one year old [48]. Infants who have undergone allo-HSCT are at a higher risk of experiencing severe adverse effects, including interstitial pneumonia syndrome and sinusoidal obstruction syndrome. The risk of relapse in this population group is significant, and a deeper study of this problem will help identify new approaches in the management of such patients [49].

Many research groups have noted late effects in the form of growth disorders and developmental delays, after treatment, especially with the inclusion of allo-HSCT. The accession of severe infectious complications requires aggressive antimicrobial prophylaxis, even outside of neutropenia. Stratification into risk groups of acute lymphoblastic leukemia in infants depending on the presence of adverse factors [50]. Based on pooled data from numerous studies, the principal prognostic factors include patient age less than six months at diagnosis, presence of *KMT2A-r*, and failure to achieve bone marrow morphological remission by day 14 [51,52,53]. It remains uncertain whether increasing the use of strategies such as allo-HSCT during first remission or intensifying current hybrid protocols that combine both lymphoid- and myeloid-targeted therapies, can improve cure rates for patients [53,54].

The experience of our center has demonstrated acceptable transplantation mortality and satisfactory OS results. Patients who received monoclonal antibodies before the allo-HSCT stage showed the best results, while it did not matter which remission the patient was in before allo-HSCT. All patients had their MRD levels assessed by flow cytometry. Of the four patients with positive pre-HSCT MRD status, two were alive and two died (one from toxic complications, the other from early relapse). Two cases of primary graft failure were associated with severe infection in the early post-transplant period, and one patient with primary graft failure underwent secondary allo-HSCT with a donor switch—he is alive and in remission now. Few GVHD cases suggest that our GVHD prevention strategies are effective and safe. 

One of the promising directions in IL therapy is the combination of allo-HSCT and CAR-T cells. Small clinical studies have shown the effectiveness of CAR-T therapy for IL and satisfactory tolerability, despite existing concerns that adverse events in infants may limit the use of this strategy [55]. CAR-T bridging to allo-HSCT has demonstrated high efficacy in the treatment of childhood ALL and can be considered as a promising treatment strategy for R/R IL [56].

Based on our limited experience in transplanting patients with infant leukemia, the overall survival is within the average values reported by other research groups. It is difficult to say for certain what the results would have been without allo-HSCT, as we did not compare groups but rather described our transplantation experience. However, the fact that only 2 out of 11 patients relapsed after allo-HSCT seems to be a good result. Speaking about complications, as in many other studies, GVHD comes to the forefront, and developing GVHD prevention programs is important. Those children who are currently alive do not have severe long-term complications that are mentioned in some studies on IL transplantation.

Can we say unequivocally that allo-HSCT in all cases of infantile leukemia is strictly necessary based on our clinical cases? Probably not. The fact that six out of eleven patients who underwent allo-HSCT are currently alive with no evidence of leukemia supports the efficacy of allo-HSCT in this patient category. Against allo-HSCT; however, is the fact that some of the surviving patients suffer from cGVHD—a condition that severely affects the child and may be life-threatening. Furthermore, two patients died in the early post-transplantation period due to specific allo-HSCT complications; perhaps they would have survived using a standard chemotherapeutic approach. The results obtained in this study generally correlate with the data from previous clinical trials and demonstrate the efficacy of allo-HSCT in the treatment of relapsed/refractory infant leukemia. However, significant limiting factors include the toxicity of conditioning regimens and the development of GVHD.

These factors must be considered when selecting conditioning regimens, as infants are at high risk of toxic effects from myeloablative chemotherapy regimens and complications of allo-HSCT. Therefore, the decision to perform allo-HSCT for R/R IL requires careful patient selection [57]. A recent study has shown that allo-HSCT is more effective in preventing disease relapses in infants with MRD-negative status. Reduced-intensity conditioning regimens are also considered beneficial for mitigating toxic effects [58]. To prevent GVHD in infants, it is necessary to perform depletion of αβ T-cells in the graft. To reduce the severity of GVHD and improve graft engraftment, mesenchymal stem cells (MSCs) from the same donor as the hematopoietic stem cells (HSC) can be used. MSCs have repeatedly demonstrated efficacy in the treatment and prevention of GVHD and graft failure in childhood leukemia [59,60].

To develop an optimal strategy for using allo-HSCT in the treatment of infant leukemia, additional clinical studies are needed, utilizing modern methods of treatment and prevention of allo-HSCT complications.

Limitations: This study has several limitations. First, most of the studies included in analysis are retrospective observational studies, and there are no randomized controlled trials. Second, our experience includes a small patient number and a limited follow-up period.

## 7. Conclusions

In this small single-center series, allo-HSCT in infants with high-risk acute leukemia was feasible but associated with substantial early toxicity; the relapse rate appeared low at short follow-up. Larger, collaborative datasets with standardized indications, transplant platforms, and MRD-guided selection are needed to define the role of allo-HSCT in this population. It is also necessary to take into account all the ethical aspects of performing allo-HSCT on infants, understanding that this procedure often leads to disability. Better study and understanding of the genetics and biology of IL will stimulate the development of new therapeutic strategies to improve the quality of life of children in the future.

## Figures and Tables

**Table 1:** Features of a group of infants with acute leukemia who received allo-HSCT.

Feature	Number of Patients n/%
Type of acute leukemia	
AML	6/54.5%
ALL	3/27.2%
MPAL	2/18.3%
Sex distribution	
Male	6/54.5%
Female	5/45.5%
*KMT2A-r*	
Yes	6/54.5%
No	5/45.5%
Initial CNS-status	
Positive	1/9.1%
Negative	10/90.9%
Status of remission	
CR 1	6/54.5%
CR 2	5/45.5%
MRD status	
Positive	4/36.4%
Negative	7/63.6%
Novel drugs	
Blinatumomab	1/9%
Inotuzumab ozogamicin	2/18.2%
Nelarabine	1/9%
No novel drugs	7/63.8%

**Table 2:** A compilation of research papers. Treatment results of IL according to current studies.

Study	Disease	Country	Period (y)	HSCT (n)	NonHSCT (n)	Results	References
Mann et al.	ALL	International	1999–2006	37	240	4-y DFS HSCT 60%; 4-y DFS chemotherapy 47%	[35]
Kosaka et al.	ALL	Japan	1998–2002	29	15	3-y EFS HSCT in CR1 64%;3-y EFS overall 44%	[36]
Sanders et al.	ALL	United States	1982–2003	40	0	3-y DFS overall 42%; 3-y DFS for HSCT in CR1 76%;	[37]
Dreyer et al.	ALL	United States	1996–2000	53	47	5-y EFS HSCT 49%; 5-y EFS chemotherapy 49%	[38]
Murray et al.	ALL	United States	1986–2005	4	5	HSCT OS 75%; Chemotherapy OS 60%	[39]
Creutzig et al.	AML	Germany	1998–2010	14	0	OS HSCT 93%	[40]
Pieters et al.	ALL	United States	2006–2024	111	494	4-year DFS after HSCT 44.0%	[41]
Parikh et al.	ALL AML MDS	International	2000–2014	472	0	3-y OS 31%	[42]
Kawasaki	AML	Japan	1995–1998	2	26	EFS HSCT 100%; EFS chemotherapy 77%	[43]
Takachi T	ALL	Japan	2019	43	13	13 pts relapsed after HSCT,1 pt died in CR, and 29 pts are in CR	[44]
Jacobsohn et al.	ALL	United States	1992–2005	16	0	4-y EFS 75%	[45]
Wang et al.	AML	China	2013–2022	27		3-y DFS after HSCT of 28%	[46]

**Table 3:** Outcomes of allo-HSCT patients with IL at the NN Blokhin National Medical Research Center of Oncology performed between 2021 and 2023. (Median follow-up was 23.5 months).

	Donors	MuRD (n)	MUD (n)	Haplo (5\10 HLA) (n)
Feature				
Cases		1	5	5
Type IL		AML	AML (2) ALL (2) MPAL (1)	AML (3) ALL (1) MPAL (1)
Regimen		treosulfan + fludarabine + thiotepa	treosulfan + fludarabine + thiotepa (3) busulfan + fludarabine +melphalan (2) (ALL)	treosulfan + fludarabine + thiotepa (2) treosulfan + fludarabine + melphalan (2) busulfan+cytarabine +cyclophosphamide (1) (ALL)
PTCy		No	No	3
TCRαβ/CD19 depletion		No	No	2
aGVHD		No	aGVHD (grade I–II) (2) aGVHD (grade III–IV) (1)	aGVHD (grade III–IV) (2)
cGVHD		No	extensive form(1)	extensive form(1)
Transplant complications		Treosulfan toxicoderma grade 2 Immune fever	Febrile fever (4) Treosulfan toxicoderma (2) Cardiomyopathy (1)	Febrile fever (3) Capillary leak (1) VOD (1)
Infections		CLABSI	Intestinal infection (4) Soft tissue infection (2) Oropharyngeal mucositis (3) BKV viremia (1)	Intestinal infection (3) Soft tissue infection (2) CMV viremia (3) ADV viremia (1) Oropharyngeal mucositis (5) Sepsis (1)
Median time to leukocytes engraftment (days)		16	13	16
Median time to platelet engraftment (days)		12	12	20
Primary graft failure		No	No	2
Post-transplant relapse		No	No	2
Survival cases (n)		1	4	1

## Abbreviations

HSCT	hematopoietic stem cell transplantation
Allo-HSCT	allogeneic hematopoietic stem cell transplantation
HSC	hematopoietic stem cells
PTCy	Post-transplant cyclophosphamide
AL	acute leukemias
AML	acute myeloid leukemia
ALL	acute lymphoblastic leukemia
MPAL	mixed-phenotype leukemia
IL Infant	acute leukemia
DNA	deoxyribonucleic acid
RNA	ribonucleic acid
miRNA	microRNA
GVHD	graft-versus-host disease
aGVHD	acute graft-versus-host disease
cGVHD	chronic graft-versus-host disease
IST	immunosuppressive therapy
ATG	antithymocyte globulin
CNS	central nervous system
BFM	Berlin–Frankfurt–Münster
FAB	French–American–British classification
MRD	minimal residual disease
MaRD	matched related donor
MUD	matched unrelated donor
MMRD	mismatched related donor
NG2	neutron glial antigen-2
NGS	next-generation sequencing
TLS	tumor lysis syndrome
COG	Children’s Oncology Group
JPLSG	The Japanese Pediatric Leukemia/Lymphoma Study Group
CLABSI	central line-associated bloodstream infection
EFS	event-free survival
TBI	total body irradiation
qPCR	real-time quantitative PCR
CAR-T	chimeric antigen receptor therapy
OS	overall survival
DFS	disease-free survival
IQR	interquartile range
MAGIC	Mount Sinai Acute GVHD International Consortium CLABSI—central line-associated bloodstream infection
CMV	cytomegalovirus
BKV	BK virus
ADV	adenovirus
VOD	veno-occlusive disease
R/R	relapsed/refractory
MSCs	mesenchymal stem cells
CR	complete remission
NIH	National Institutes of Health
